# Efficacy of antimicrobial and anti-viral coated air filters to prevent the spread of airborne pathogens

**DOI:** 10.1038/s41598-022-06579-9

**Published:** 2022-03-09

**Authors:** Rowan Watson, Morwenna Oldfield, Jack A. Bryant, Lily Riordan, Harriet J. Hill, Julie A. Watts, Morgan R. Alexander, Michael J. Cox, Zania Stamataki, David J. Scurr, Felicity de Cogan

**Affiliations:** 1 Institute of Microbiology and Infection, University of Birmingham, Birmingham, UK; 2 Institute of Immunology and Immunotherapy, University of Birmingham, Birmingham, UK; 3 Advanced Materials and Healthcare Technologies Division, School of Pharmacy, University of Nottingham, Nottingham, UK

**Keywords:** Antimicrobials, Applied microbiology, Environmental microbiology, Microbiology, Materials science

## Abstract

The COVID-19 pandemic has demonstrated the real need for mechanisms to control the spread of airborne respiratory pathogens. Thus, preventing the spread of disease from pathogens has come to the forefront of the public consciousness. This has brought an increasing demand for novel technologies to prioritise clean air. In this study we report on the efficacy of novel biocide treated filters and their antimicrobial activity against bacteria, fungi and viruses. The antimicrobial filters reported here are shown to kill pathogens, such as *Candida albicans*, *Escherichia coli* and MRSA in under 15 min and to destroy SARS-CoV-2 viral particles in under 30 s following contact with the filter. Through air flow rate testing, light microscopy and SEM, the filters are shown to maintain their structure and filtration function. Further to this, the filters are shown to be extremely durable and to maintain antimicrobial activity throughout the operational lifetime of the product. Lastly, the filters have been tested in field trials onboard the UK rail network, showing excellent efficacy in reducing the burden of microbial species colonising the air conditioning system.

## Introduction

The risk of respiratory infection is increased in people using public transport and was noted to be sixfold higher in people who had used a tram or bus within 5 days of the onset of symptoms^1^. Investigation of London Underground Oyster card data demonstrated a significant correlation between higher rates of influenza-like illnesses in people with increased underground usage and/or those who incur more contact while travelling in London boroughs^2^. The risk of developing COVID-19 is also increased with greater public transport use by several studies^3^. In particular, a recent case study demonstrated that among the 68 passengers who travelled on a bus with one infected individual, 24 became infected with SARS-CoV2. This is compared to none who travelled on a separate bus^4^. Both buses had air conditioning on a recycling mode, and the pattern of spread supported a role for airborne transmission, as the risk of COVID-19 was not significantly associated with distance from the index case.

There have been a number of novel filters introduced to try and prevent the spread of viruses through heating, ventilation and air conditioning (HVAC) systems. For example, in aerospace cabins HVAC systems are designed to be used with high-efficiency particulate air (HEPA) filters^5^. HEPA are extremely efficacious at screening out airborne viruses and bacteria due to their small particulate size^6^. However, a significantly higher level of energy is required to push air through HEPA filters compared to basic HVAC filters. While these air filtration systems can effectively remove pathogenic microorganisms from the indoor air environment, the organisms are not destroyed and can remain viable, or even proliferate, within the air filtration system and on the filter media. Therefore the filter itself can behave as a source for contamination of the air environment with airborne pathogenic microorganisms^7,8^.

Due to the risk of filters acting as a reservoir for contamination of the indoor air environment, and in response to the COVID-19 pandemic, there has been an increase in demand for novel antimicrobial technologies to prevent transmission. The incorporation of silver nanoparticles into meshes for filters has also been tested in a range of forms^9–11^. Dong et al. reported the development of a nanofibrous air filter with incorporated silver nanoparticles to impart an antimicrobial effect^12^. The inclusion of silver nanoparticles demonstrates an effect on survival of both gram-positive and gram-negative bacteria on the filters. The effect appeared to be bacteriostatic with no growth observed over the 24 h, rather than bactericidal. Limited efficacy of the filters was also demonstrated against coronaviruses, however the reduced survival effect falls short of the required log reduction needed for it to be registered as a biocidal product^13^. Alternative approaches include a novel carbon nanofiber technology, which used particles of specific size similar to bacteria and viruses to demonstrate enhanced airborne contamination capture^14^. It is not known if this will translate to be efficacious against bacteria and viruses as well as particle modelling. However, Park et al. have successfully demonstrated that the inclusion of carbon nanotubes onto glass filters can reduce the survival of viruses after contact with the filter^15^. Carbon nanotube filters demonstrated a 90% reduction in virus survival downstream of the filter, which is significantly more effective than the silver nanoparticle technology. While these technologies require development of new filters, existing filters can also be modified with biocides, as reported by Kim et al., in which HEPA filters are modified with tannic acid^16^. The modified air filters demonstrated ~ 90% capture of the H1N1 virus compared to standard HEPA filters.

In this work, we take the approach of modifying existing filter technologies with a broad range biocide, chlorhexidine digluconate (CHDG). We describe the efficacy of a filter where the filter fibres have been coated with CHDG in order to reduce the microbial reservoir on the filter for re-contamination of the air environment. The stability and durability of the filter is examined and the antimicrobial efficacy against gram-positive bacteria, gram-negative bacteria, fungi and viruses is assessed.

## Results

### Characterisation of CHDG modified filters

**Figure 1:**
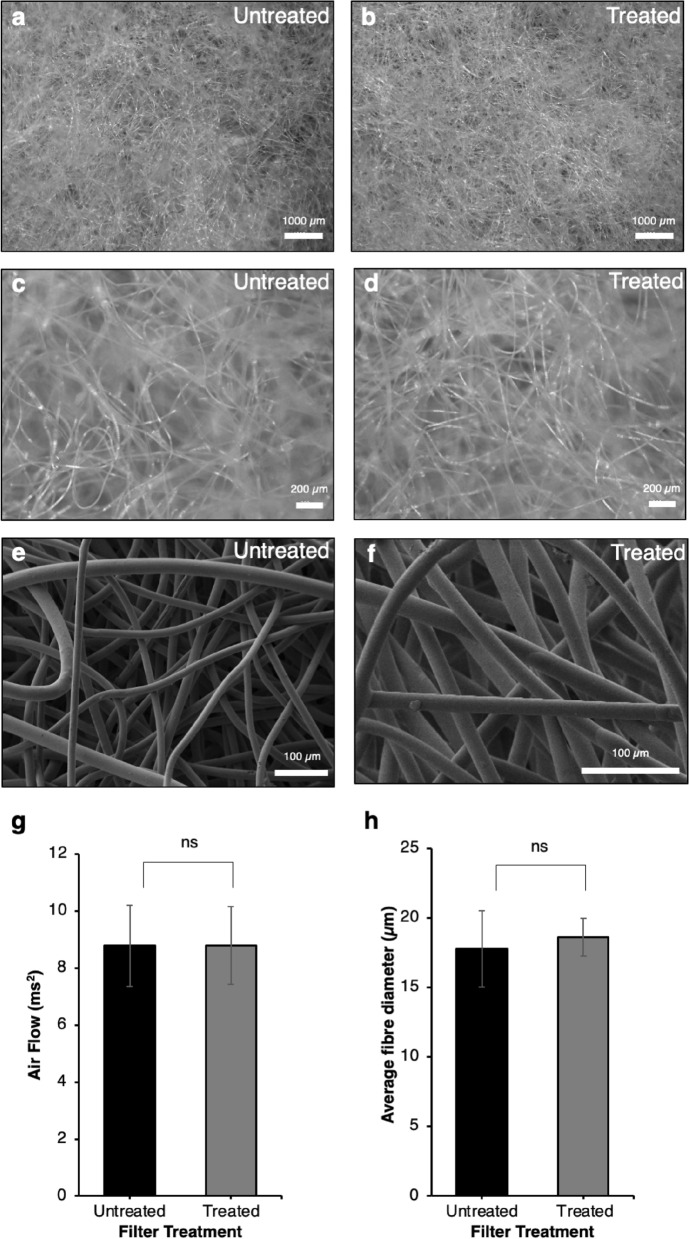
Characterisation of CHDG treated filters. (**a**) Light microscopy of an MK3 filter at ×3.5 magnification. Image is representative of all images, scale bar 1000 μm. (**b**) Light microscopy of treated antimicrobial filter at ×3.5 magnification. Image is representative of all images, scale bar 1000 μm. (**c**) Light microscopy of an MK3 filter at ×64 magnification. Image is representative of all images, scale bar 200 μm. (**d**) Light microscopy of treated antimicrobial filter at ×64 magnification. Image is representative of all images, scale bar 200 μm. (**e**) low magnification SEM images of MK3 filter, image is representative of all images, scale bar 100 μm (**f**) SEM images of antimicrobial filter, image is representative of all images, scale bar 100 μm (**g**) Graph shows the average rate of air flow through standard and antimicrobial filters, n = 12, error bars show standard error of the mean. (**h**) Graph shows the average fibre diameter of untreated and treated filters, measured from SEM images, n = 51, error bars show standard error of the mean.

There is an urgent need for technologies which can eliminate the reservoir of microorganisms on air filters and thus decrease airborne transmission of disease. We have previously shown that incorporation of biocides onto non-porous materials can demonstrate excellent antimicrobial efficacy against a range of organisms^17^. In this work we have expanded this technology to demonstrate the efficacy of biocides when incorporated into fibrous materials. MK3 filters (supplied by Pullman AC) were modified by NitroPep Ltd. in order to incorporate CHDG into the filter media. Both the standard MK3 filters and CHDG treated MK3 filters were imaged using light microscopy and SEM to determine visible and structural differences between the standard MK3 filters and the CHDG treated MK3 filters (Fig. 1a–d). Images at both 3.5× and 64× magnification showed no structural differences between the control and treated filters following incorporation of the biocide. Imaging was also carried out using SEM. SEM images also demonstrated no visible differences at either magnification (Fig. 1e,f).

The effect of CHDG incorporation on air flow through the filter was then examined to ensure no changes in the efficiency of air flow in the system. Filters were installed in an industrial condensing unit and the air flow across both standard and biocide treated filters was measured (Fig. 1g). The air flow across the control filter was 8.79 ± 1.42 m/s and the CHDG treated filter was 8.79 ± 1.35 m/s, demonstrating there was no significant difference from the control filter and that CHDG treatment did not impede air flow through the filter. This result is in agreement with the SEM data, which demonstrated that the average fibre diameter was unchanged by treatment of the filter (Fig. 1h).

**Figure 2:**
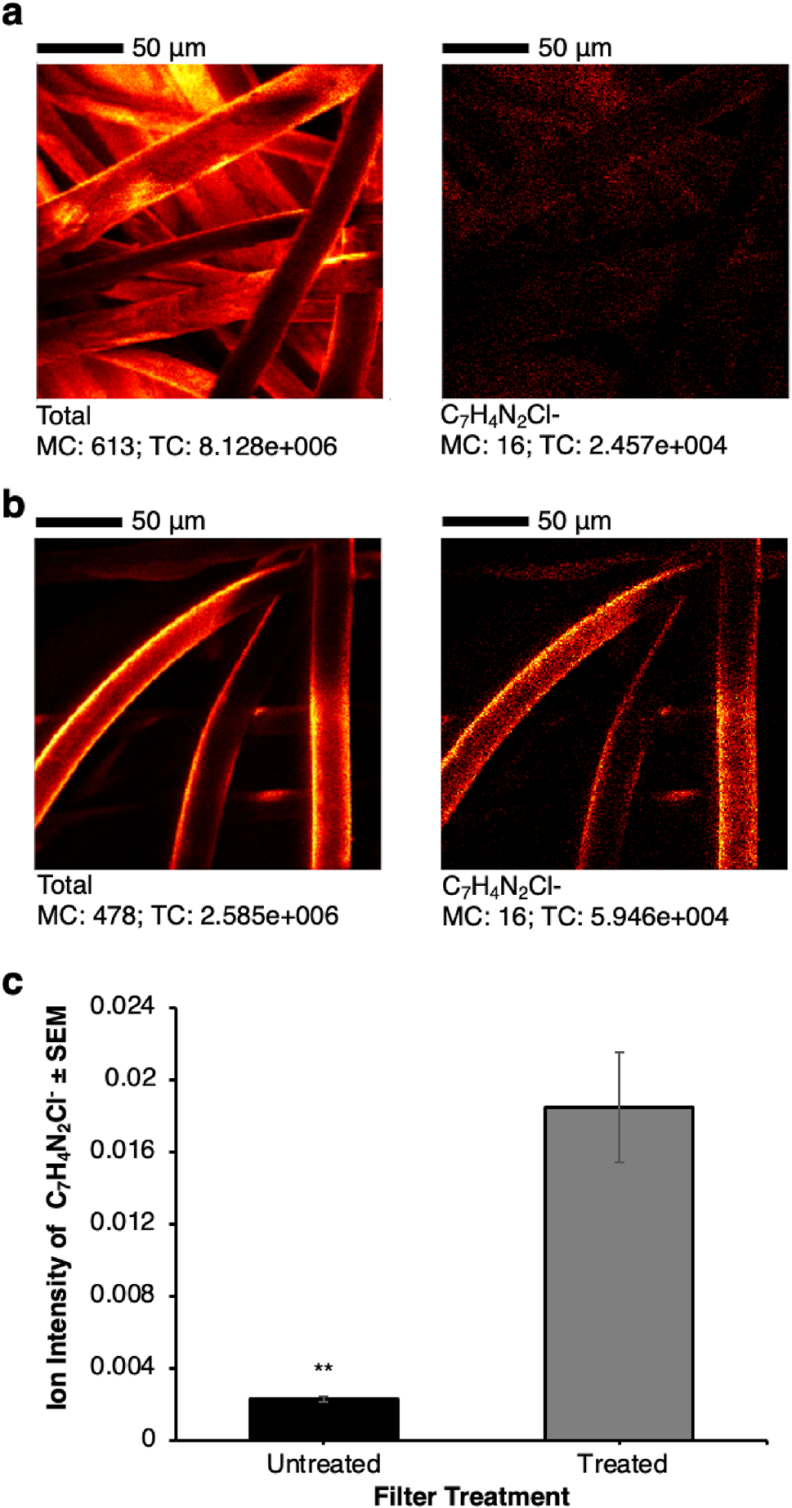
Characterisation of CHDG treated filters by ToF SIMS. (**a**) ToF SIMS of an untreated MK3 filter. Image is representative of all images, scale bar 1000 μm. (**b**) ToF SIMS of a treated MK3 filter. Image is representative of all images. (**c**) Graph shows the average ion intensity for control and treated filters, n = 4, error bars show standard error of the mean, ** denote statistical significance p < 0.01.

In order to confirm that the surface of the filter fibres was chemically modified using CHDG, 3D OrbiSIMS analysis was performed upon the untreated and treated filters using the C_7_H_4_N_2_Cl^−^ secondary ion. This secondary ion has been previously established as being diagnostic of the CHDG molecule^18^ and was shown here to be absent in the untreated filter data. The distribution of CHDG coverage was observed to be on all fibres in the filter and to be uniformly distributed across the fibre surfaces (Fig. 2a,b). The variation in distribution upon fibres observed is proposed to be the result of a combination of sample topography and shadowing where secondary ions are collected less effectively in some areas. This is confirmed with the C_7_H_4_N_2_Cl^−^ secondary ion distribution correlating with intensity variations within the total secondary ions. The relative amount of chlorhexidine applied to the fibre surfaces is illustrated in Fig. 2c which shows the C_7_H_4_N_2_Cl^−^ secondary ion intensity for untreated and treated samples.

### Antimicrobial efficacy of CHDG filters

**Figure 3:**
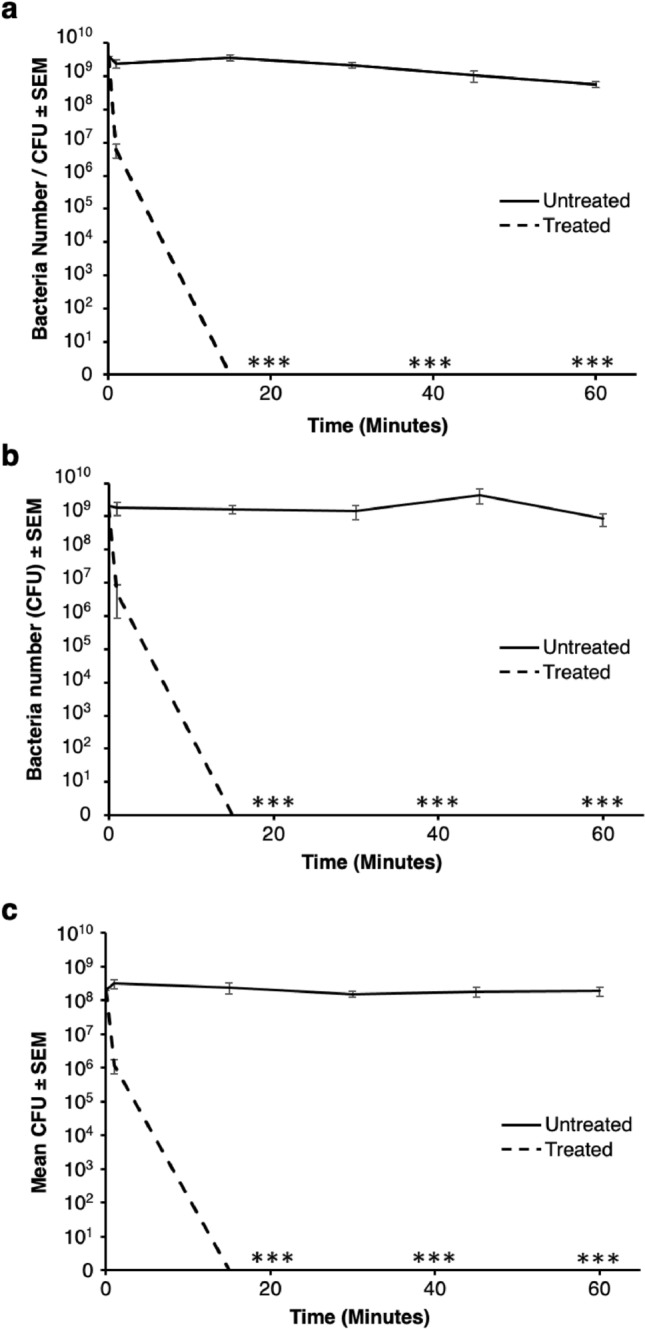
Antimicrobial efficacy of filters. (**a**) Time course showing *E. coli* survival at different time points following incubation on untreated and CHDG treated MK3 filters. *** denotes statistical significance < 0.001, n = 9. Error bars show standard error of the mean. (**b**) Time course showing MRSA survival at different time points following incubation on untreated and CHDG treated MK3 filters. *** denotes statistical significance < 0.001, n = 9. Error bars show standard error of the mean. (**c**) Time course showing *C. albicans* survival at different time points following incubation on untreated and CHDG treated MK3 filters. *** denotes statistical significance < 0.001, n = 9. Error bars show standard error of the mean.

Having established that the filters could be treated with CHDG without impeding the air flow through the filter or the structure, the antimicrobial efficacy of the filters was tested against the model gram-negative bacterium, *Escherichia coli*. Killing was observed over 1 h with time points at 1, 15, 30, 45 and 60 min. Cells were added to the surface and recovered from the surfaces at each time point for counting of CFU (Fig. 3a). Survival of the cells was decreased at each time point, with a 3-log reduction observed at 60 s on the CHDG treated samples and no recoverable cells at any further time point. This is significantly lower than the control samples, which had no significant effect on bacterial survival over the entire time period. This trend was also observed when the filters were tested against the model gram-positive bacteria, *Staphylococcus aureus* (Fig. 3b). The number of cells surviving on the surface decreased rapidly with 4 × 10^6^ ± 3 × 10^6^ cells surviving at 1 min and no cells recoverable from the surfaces in the time points following 1 min. This was significantly lower than control surfaces which had no effect on bacterial survival over the 1 h time period.

After the efficacy against bacteria was determined, the filters were exposed to fungi using the model organism *Candida albicans*. Using the same protocol and time points as that used for experiments with bacteria, the filters were exposed to *C. albicans* (Fig. 3c). The filters showed a significant antifungal effect. The number of cells surviving after 60 s was 4 × 10^6^, after which no cells were recovered at any other time point. This demonstrated that the filters are antifungal as well as antibacterial.

**Figure 4:**
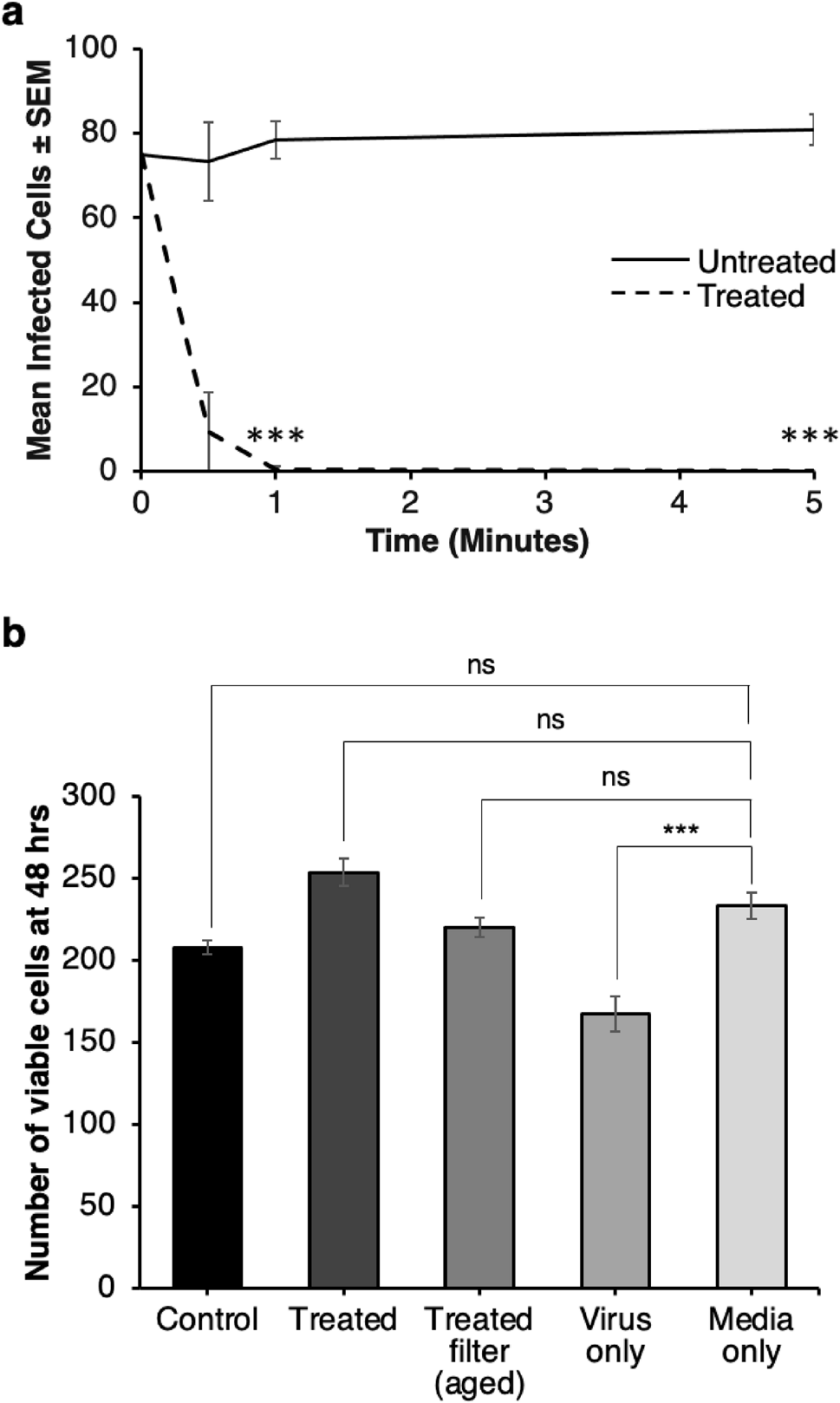
Antiviral efficacy of filters. (**a**) Time course showing SARS-CoV-2 inactivation at different time points following incubation on untreated and CHDG treated MK3 filters. *** denotes statistical significance < 0.001, n = 9) (**b**) Effect of filters on viability of Vero cells per field of view. 6 fields of view per well. Wells analysed in triplicate. n = 2. Error bars show standard error of the mean. ***p < 0.0005.

Finally, to be able to determine the full extent of the antimicrobial nature of CHDG treated filters, they were tested for antiviral efficacy against SARS-CoV-2 (Wuhan strain). 10^4^ infectious units (IU) were applied to the filter surfaces and incubated for time periods up to 5 min. Following the incubation period, viral particles were lifted from the filter surface using cell culture media and incubated with Vero cells to determine the capacity of remaining virions to infect target cells (Fig. 4a). The treated filters demonstrated excellent virion inactivation rates with the number of infected cells being decreased from 80 ± 3% from the control filters to 9 ± 9% on the treated filters after 30 s exposure to the filters and reducing further to 0 infected cells after 1 min incubation on the treated filters. Some toxicity of the viral suspension on the Vero cells was also observed (Fig. 4b). The extract from the filters triggered no decrease in cell number or survival from any of the filters. The only samples which demonstrated significant reduction in Vero cell numbers were those in which the SARS-CoV-2 virus was added directly to the cells.

### Durability of CHDG treated filters

**Figure 5:**
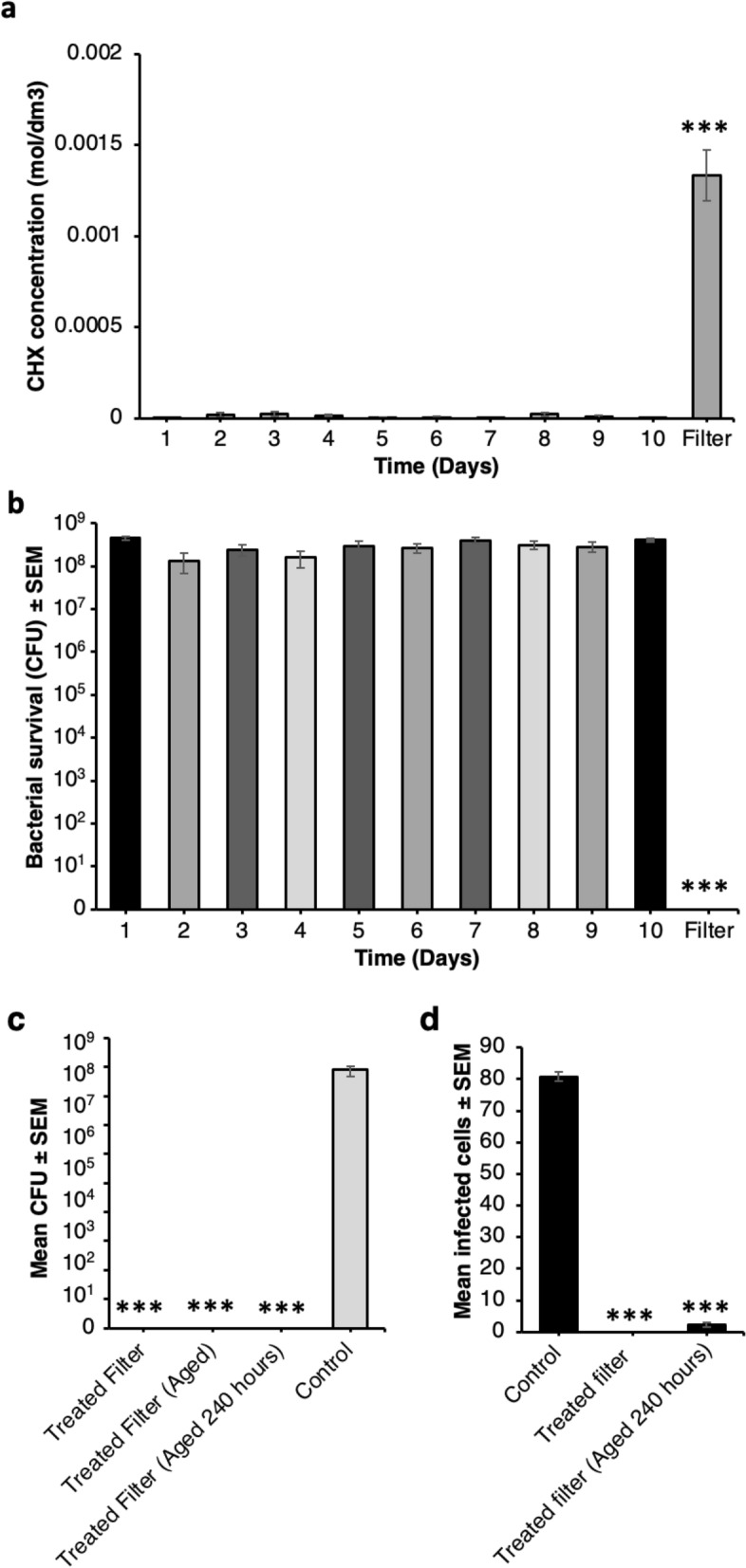
Filter durability. (**a**) Mean concentration of chlorhexidine leached from the filters over time and the concentration of chlorhexidine remaining in the filter at the end of durability testing measured using an absorbance assay. Error bars show standard error of the mean, n = 9, *** denotes statistical significance p < 0.001. (**b**) Mean bacterial survival after incubation in chlorhexidine extract from durability filter test measured in (**a**). Error bars show standard error of the mean, n = 9, *** denotes statistical significance p < 0.001. (**c**) Antimicrobial efficacy of filters before and after durability testing against *E. coli*. Error bars show standard error of the mean, n = 9, *** denotes statistical significance p < 0.001. (**d**) Antiviral efficacy of filters before and after durability testing against SARS-CoV-2. Error bars show standard error of the mean, n = 9, *** denotes statistical significance p < 0.001.

For the filters to be effective they should demonstrate durability and minimal release of biocides. In order to determine the durability of the antimicrobial on the filter, filters were subjected to two different durability tests. Firstly, the filters were sectioned and installed in a laboratory test rig complete with a capture membrane to determine if any CHDG leaches from the surfaces due to air flow. The filters were maintained in air flow continuously for 240 h. The capture membrane was removed and analysed for biocide at 24 h intervals and potential leached CHDG was then released from the membrane into solution. The amount of CHDG in the solution was monitored using an established assay^19^. No measurable amount of CHDG was leached from the filter throughout the duration of the test (Fig. 5a). Following the final time point of the durability test, the filter was destroyed and CHDG extracted. The remaining CHDG in the filter after 240 h was measured at 82% ± 15% of the applied CHDG. This demonstrates that the biocidal treatment is very stable and confirms the results of the air flow durability study.

Extracted CHDG solutions from the durability test and the time-course interval filters were used to test bacterial survival as *E. coli* is sensitive to significantly lower concentrations of CHDG than that detectable by absorbance/fluorescence assays (*E. coli* K-12 BW25113 CHDG MIC = 2 µM). Extract from the membranes was incubated with 1 × 10^6^ CFU *E. coli* for 1 h and bacterial survival was assessed by dilution and counting of CFU (Fig. 5b). Despite this assay being sensitive to increases in concentration of 2 µM, no significant changes in bacterial survival were observed from the extract of membranes from any time point. When CHDG was extracted from the filter by destruction after the final time point, no CFU were recoverable from the *E. coli* survival assay, confirming that the filters are durable throughout their lifecycle and do not leach significant amounts of CHDG.

To assess the chemical stability of the filters, their antimicrobial efficacy against *E. coli* and SARS-CoV-2 was tested following aging through the durability assay. Filters were aged in the 240 h durability test, or by installation in a full air condensing unit for 6 h then tested for antimicrobial efficacy against *E. coli* (Fig. 5c). The filters were tested as described for the antimicrobial efficacy tests and bacteria were incubated on the filter for 30 min. The *E. coli* survived well on untreated filters, with CFU of 7.9 × 10^8^ ± 3.5 × 10^8^, however there were no recoverable bacteria on treated filters aged by both methods, which was comparable to unaged filters. This pattern was also observed when aged filters were tested against SARS-CoV-2 (Fig. 5d). Viral particles maintained the ability to infect mammalian cells after incubation on the standard filters, whereas following incubation on the treated filters, no infection was observed. After the filters were aged there was no significant increase in infection of the cells compared to the unaged filter.

**Figure 6:**
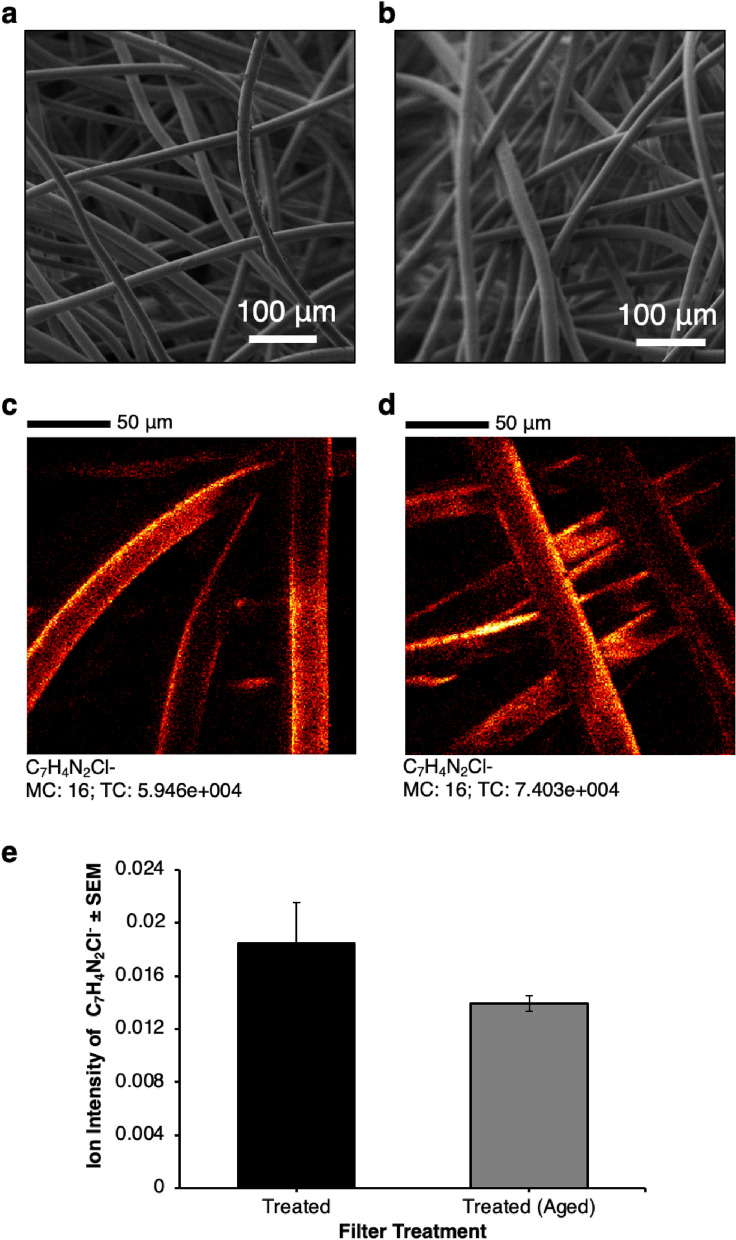
Characterisation of filters following aging. (**a**) SEM images of antimicrobial treated filter, image is representative of all images, scale bar 100 μm. (**b**) SEM images of antimicrobial filter after ageing, image is representative of all images, scale bar 100 μm. (**c**) ToF SIMS of a treated filter. Image is representative of all images, scale bar 50 μm. (**d**) ToF SIMS of an antimicrobial treated and aged filter. Image is representative of all images, scale bar 50 μm. (**e**) Graph shows the average ion intensity for treated filters before and after ageing, n = 4, error bars show standard error of the mean.

To assess the morphological stability of the filters, the treated filters aged for 240 h were imaged using SEM (Fig. 6a,b). The images showed no differences between the treated filters before and after the durability process. ToF–SIMS was also carried out on treated filters before and after durability testing (Fig. 6c,d). The images showed no apparent differences to the CHDG coatings on the fibres after the ageing process, which is likely why the filters retained antibacterial and antiviral activity after aging. The ion intensity was quantified and no significant differences were seen between the treated filter before and after the durability testing (Fig. 6e).

### Efficacy of CHDG treated filters in an operational environment

**Figure 7:**
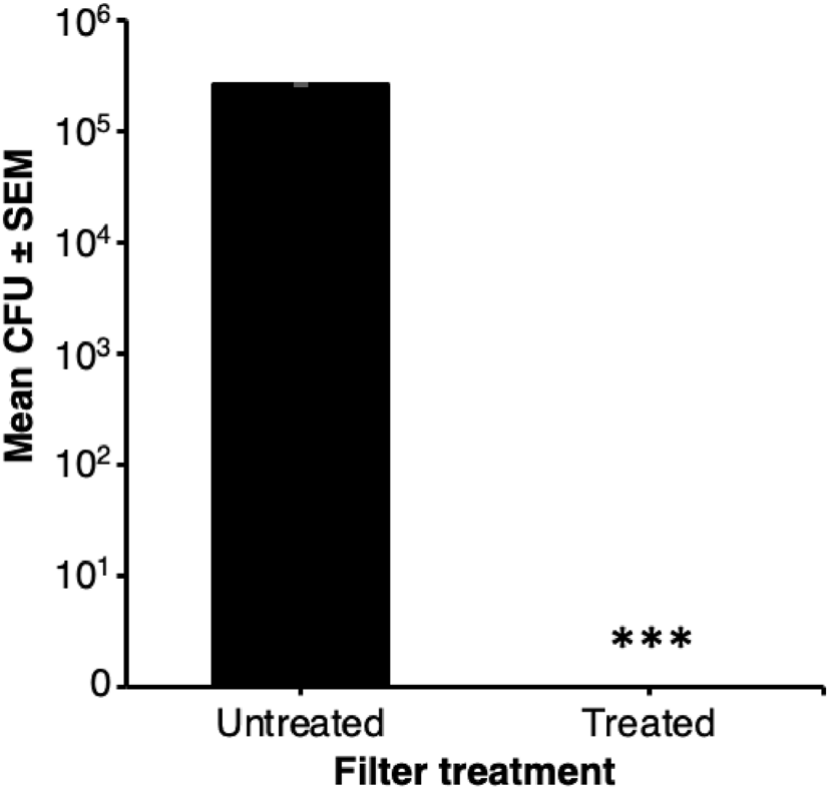
Efficacy of filters following installation on trains on the UK rail network for 3 months. Mean CFU were calculated for the entire filter based on the size of the sample filter for extraction. Error bars show standard error of the mean. ***p < 0.001, n = 3.

In order to determine the efficacy of the filters in an operational environment, standard and treated filters were installed in carriages on trains in the UK (Fig. 7). The filters were installed for 3 months in matched pairs across carriages on the same train line. After their life cycle, filters were removed and microorganisms were extracted in order to quantify culturable organisms by CFU counting. All standard filters had > 2 × 10^6^ CFU on the filter, whereas the treated filters had no detectable bacterial or fungal organisms. This supports the laboratory studies and paves the way for the use of air filters in public spaces as a method of reducing the microbial community colonising air filtration systems and reducing the spread of airborne pathogens.

## Discussion

Previous studies have demonstrated that the spread of airborne disease is possible on closed systems such as public transport^4,20^. It is well documented that the increase of ventilation can significantly reduce the spread of disease in such areas. However, before the COVID-19 pandemic commenced in 2020 the driving force in HVAC was to have closed systems in order to reduce energy wastage, which in turn limited ventilation possibilities. Train HVAC systems recycle up to 90% of the air in a closed environment and the filters are only designed to remove particles, such as pollution and pollen (6–100 microns), not to inactivate pathogenic microorganisms^21^. This is in contrast to air transport systems, which use HEPA filters (effective from 0.01 micron) to clean the air before recycling it. Despite the target size range for particle filtration being greater than many microorganisms, the filters within HVAC systems have been shown to harbour potentially harmful microorganisms such as bacteria, fungi and viruses that can lead to subsequent pollution of the air environment^22–24^.

There have been multiple attempts to generate efficient antimicrobial air filters to allow standard air filtration systems to remove and inactivate pathogens from air. The most commonly used antimicrobial in the generation of air filters is silver. Silver has been incorporated in a number of different ways through the incorporation of silver/carbon nanotubes into the filter material^25^, the incorporation of chitosan, silver and silica nanoparticles into the filter material^26^ or through the generation of a composite fibre of poly(vinyl alcohol)/poly(acrylic acid) loaded with silver nanoparticles^27^. While these technologies are effective, they take > 18 h to reduce bacterial load. This is a significant drawback, as the nature of HVAC systems requires that the pathogens be killed in seconds as they pass through the filter. As we have demonstrated, there is a high level of microorganism survival on the filter material for extend periods of time, meaning that contaminated filters could behave as a reservoir for pathogens to be dislodged from the filter causing an infection risk hours or days later. The treated filters described in this paper show antimicrobial efficacy in under 60 s against bacteria, fungi and viruses therefore addressing this issue and limiting the potential for re-contamination of the air environment.

The speed of antimicrobial efficacy of this technology is likely due to the incorporation of CHDG as the bioactive molecule, which is a cationic bisbiguanide that is a broad spectrum antiseptic. While the precise molecular mechanisms of action against the different organisms used in this study are unknown, CHDG acts through the disruption of cellular membranes, which is why it is also active against enveloped viruses, such as the SARS-CoV-2 virus used in this study, but not non-enveloped viruses^28^. This broad mechanism of action means that this technology will be active against a wide range of microorganisms found to contaminate HVAC systems^4,20^, as demonstrated by the select model organisms used in this study.

The durability of the filters is also an essential component of the system. The COVID-19 pandemic has seen the rise of multiple new technologies capable of “purifying” the air such as the installation of equipment containing UV light or the use of HEPA filters^29^. Neither of these is ideal for the majority of existing HVAC systems as they both require significant infrastructure upgrades. In comparison, the technology we report here can be applied to existing filters and used in existing HVAC systems with no modifications. Unlike the novel polymer materials, treatment of filters with CHDG can be applied to any porous filter. Therefore, the base filter material does not need to be modified and the treatment can be applied to all filters currently in use in HVAC systems^29^. We have also demonstrated that the CHDG treatment does not impede the existing air flow or fibre diameter of the filters when used in industrial air condensing units and that the technology is durable. This level of compatibility with existing systems removes many of the barriers encountered when new technologies are brought onto the market.

## Conclusions

We have demonstrated a novel antimicrobial treatment for air filters which is applicable to porous filters across the HVAC sector. Specifically, we have demonstrated that the treatment has no detrimental effect on rate of air-flow or fibre size of the filter, while also showing excellent antimicrobial efficacy against gram-positive and gram-negative bacteria, fungi and viruses. The filter treatments are durable throughout the existing maintenance life cycle of the filter and have been demonstrated to work in an operational environment.

## Materials and methods

All consumables were ordered from Sigma Aldrich unless otherwise stated. MK3 Filter samples were sourced from Pullman AC and pre-treated with a Chlorhexidine digluconate 0.002% solution (Azelias), by NitroPep Ltd using an automated treatment system at room temperature to produce coated filters. All reagents were purchased from Sigma Aldrich unless otherwise stated.

### Microscopy of filters

MK3 filters and CHDG coated MK3 filters were imaged using a Zeiss AxioZoom.V16 with top illumination using an LED ring and a Zeiss Axiocam 503 mono camera. Each filter was washed 3 times in sterile type 3 water and dried in a 37 °C incubator overnight. After drying, each filter was imaged again at 3.5× and 64× magnifications, back and front, and in triplicate for each condition with exposure time maintained between samples.

### Scanning electron microscopy of filters

Each filter was washed 3 times in sterile type 3 water and dried in a 37 °C incubator overnight. After drying, each filter was imaged both back and front, and in triplicate for each condition. Scanning electron microscopy was performed at an operating voltage of 1 kV, 100pA, SESI detector, using a field emission SEM (Zeiss CrossBeam 550, Oberkochen, Germany) on uncoated filter samples: local charge compensation was achieved using a nitrogen flood gun.

### Air flow rate

MK3 filters and CHDG treated filters were installed in an industrial condensing air unit and run for 6 h. The air flow rate was measured across the entire surface area of the filter using 12 coils each with an area of 0.1617 m/sq.

### 3D OrbiSIMS

Mass spectrometry imaging was performed using a HybridSIMS instrument (ION-TOF GmbH) employing a 30 keV Bi_3_^+^ primary ion source (0.3 pA target current). In order to best accommodate sample topography, delayed extraction was used for analysis with nitrogen gas flooding. Data was acquired over a 200 × 200 µm^2^ area, with a resolution of 256 × 256 pixels with 20 shots per pixel. The data acquisition and analysis was performed with SurfaceLab7 (ION-TOF GmbH). Charge neutralisation was performed using a relatively low energy (< 2 eV) electron floodgun.

### Antimicrobial efficacy testing of filters against bacteria

Both *E. coli* K-12 BW25113 and *S. aureus* ATCC6538 were cultured in 5 ml LB broth overnight at 37 µC for ~ 18 h with shaking (180 rpm) before being adjusted to ~ 1 × 10^9^ CFU/mL. Bacterial suspensions were arrayed in a 3 × 3 grid of 1 µL aliquots onto 3 replicate sample filters for each condition. Samples were incubated at room temperature for either 1, 15, 30, 45 and 60 min. Following incubation, bacteria were recovered from the samples by vortexing for 1 min in 10 mL sterile Dey-Engley’s neutralising broth with 7–10 sterile zirconium oxide beads. The neutralising broth suspension was then serially diluted in sterile PBS and survival assessed by counting CFU after incubation for 18–24 h at 37 °C on LB agar. CFU in the original suspension were calculated.

### Antimicrobial efficacy testing of filters against *C. albicans*

The *C. albicans* SC5314 isolate was kindly provided by the host–pathogen interaction research group at the University of Birmingham, UK. Single colonies of *C. albicans* were incubated in potato dextrose broth at 30 µC for ~ 18 h with shaking (180 rpm) before being adjusted to ~ 1 × 10^8^ CFU/mL. Antimicrobial efficacy of filters was tested exactly as described for the bacterial samples except for incubation on potato dextrose agar at 30 µC prior to CFU counting.

### Antimicrobial efficacy testing against SARS-CoV-2

SARS-CoV-2-England 2 (Wuhan strain) virus at 10^6^ IU/mL (GSAID Accession ID EPI_ISL_407073) was a kind gift from Christine Bruce, Public Health England. Untreated MK3 and CHDG treated filters were placed in separate 2 mL cryovials and 10 µL of viral stock added to each. The samples were incubated for 0.5, 1 and 5 min at room temperature. 200 µL of viral culture media (DMEM) was then added to each cryovial and washed gently. 50 µL of the supernatant was incubated in separate wells in a black 96 well flat-bottomed polystyrene imaging plate (Greiner) seeded with 4 × 10^4^ Vero cells. The Vero cells were cultured in DMEM supplemented with 10% foetal bovine serum, 2 mM l-glutamine, 100 U/mL penicillin, 10 µg/mL streptomycin and 1% non-essential amino acids (culture media) for 48 h at 37 °C. The cells were maintained at 37 °C and 5% CO_2_. Finally, the media was removed and discarded and the cells were fixed with ice-cold methanol for 5 min. Vero cells were washed in PBS and stained with rabbit anti-SARS-CoV-2 spike protein, subunit 1 (CR3022, The Native Antigen Company), detected by Alexa Fluor 555-conjugated goat anti-rabbit IgG secondary antibody (Invitrogen, Thermo Fisher Scientific). Cell nuclei were stained with Hoechst 33342 (Thermo Fisher Scientific). Cells were washed with PBS and then imaged and analysed using a Thermo Scientific CellInsight CX5 High-Content Screening (HCS) platform. Infected cells and cell viability were detected by measuring perinuclear fluorescence above a set threshold determined by positive (untreated) and negative (uninfected) controls. Automated quantification algorithms were developed with assistance from Dr Henri Huppert, Thermo Fisher Scientific, UK.

### Filter durability

Filter durability was tested by installing filters in a condensing unit, which was operated at a rate of 31,653 m/h for 6 h. The filters were then removed and their antimicrobial efficacy tested as described above. To determine if any leaching occurred from the filter surfaces, filters were set up with a continuous air flow of 0.5 m/s from one side of the filter to the other. On the other side of the filter, filter paper was held in close proximity to the filter with air flow passing from the filter directly through both filter and then filter paper. The filter paper was replaced every 24 h for 10 days. After 10 days, the filter was removed and stored in the dark. This process was repeated in triplicate. To determine the levels of chlorhexidine present, the filter paper and the filters were physically broken up with tweezers and incubated in deionised water for 16 h. The filters and the filter papers were then removed and stored. 50 µL aliquots of filter extract were pipetted into a sterile black-welled, black-bottomed tissue culture treated 96 well plate in triplicate with HPLC grade water, and 20% CHDG included for standards. Fluorescence was measured on a FLUOstar Omega plate reader at excitation 280 nm. The filter extract was then inoculated with *E. coli*. Overnight cultures were diluted (in LB broth) to ~ 1 × 10^8^ CFU/mL in a total of 1 mµ CHDG filter extract. The samples were incubated for 1 h at room temperature and then serially diluted in Dey-Engley’s neutralising solution before incubation on LB agar for 18–24 h at 37 °C. CFU in the original suspension were calculated.

### Efficacy of antimicrobial filters in HVAC systems on board trains

Untreated MK3 and CHDG treated MK3 filters were installed on board trains running on the UK rail network in standard HVAC systems. The filters were used in the system for the entire 3 month duration of the filter maintenance cycle on the trains. Following removal from the HVAC system, filters were sealed into separate containers and shipped for analysis. Strips of the filters were removed from both control and treated filters and incubated in Dey-Engley’s neutralising solution and the filter material destroyed physically to extract microorganisms. 100 µL of the neutralising solution was pipetted onto 5% horse blood agar and incubated for 48 h before counting of CFU. CFU on the whole filter were then estimated based on the sample size.

### Statistical analysis

Statistical analysis was carried out using SPSS. Where two groups were compared an independent t test was used to determine statistical significance. Where more than two groups were compared a One Way ANOVA was used with a Tukey posthoc analysis or for the comparison to the control, a Dunnett posthoc analysis was used.

## Data Availability

Raw data is available on request from the corresponding author.
